# Credibility Evidence for Computational Patient Models Used in the Development of Physiological Closed-Loop Controlled Devices for Critical Care Medicine

**DOI:** 10.3389/fphys.2019.00220

**Published:** 2019-03-26

**Authors:** Bahram Parvinian, Pras Pathmanathan, Chathuri Daluwatte, Farid Yaghouby, Richard A. Gray, Sandy Weininger, Tina M. Morrison, Christopher G. Scully

**Affiliations:** Office of Science and Engineering Laboratories, Center for Devices and Radiological Health, United States Food and Drug Administration, Silver Spring, MD, United States

**Keywords:** mathematical physiological model, computational modeling and simulation testing, physiologic closed-loop control systems, model credibility evidence, medical devices

## Abstract

Physiological closed-loop controlled medical devices automatically adjust therapy delivered to a patient to adjust a measured physiological variable. In critical care scenarios, these types of devices could automate, for example, fluid resuscitation, drug delivery, mechanical ventilation, and/or anesthesia and sedation. Evidence from simulations using computational models of physiological systems can play a crucial role in the development of physiological closed-loop controlled devices; but the utility of this evidence will depend on the credibility of the computational model used. Computational models of physiological systems can be complex with numerous non-linearities, time-varying properties, and unknown parameters, which leads to challenges in model assessment. Given the wide range of potential uses of computational patient models in the design and evaluation of physiological closed-loop controlled systems, and the varying risks associated with the diverse uses, the specific model as well as the necessary evidence to make a model credible for a use case may vary. In this review, we examine the various uses of computational patient models in the design and evaluation of critical care physiological closed-loop controlled systems (e.g., hemodynamic stability, mechanical ventilation, anesthetic delivery) as well as the types of evidence (e.g., verification, validation, and uncertainty quantification activities) presented to support the model for that use. We then examine and discuss how a credibility assessment framework (American Society of Mechanical Engineers Verification and Validation Subcommittee, V&V 40 Verification and Validation in Computational Modeling of Medical Devices) for medical devices can be applied to computational patient models used to test physiological closed-loop controlled systems.

## Introduction

Medical devices with PCLC technology automatically adjust therapy being delivered to a patient based on a measured physiological variable. Clinical environments such as critical care units could benefit from automated technologies because of the high number of required clinical actions (Embriaco et al., 2007; Mealer, 2016) and extensive monitoring and therapeutic devices already in use. For critically ill patients, PCLC technology may be used at the bedside to provide supportive therapy including automating fluid delivery (Kramer et al., 2008; Rinehart et al., 2014), mechanical ventilation and oxygen therapy (Morozoff and Saif, 2008; Tehrani, 2012), and/or anesthetic delivery (Dumont et al., 2009). However, the presence of control algorithms to automate therapeutic actions can introduce new hazards (Parvinian et al., 2018) and thus a comprehensive safety assessment is crucial to their success.

**FIGURE 1:**
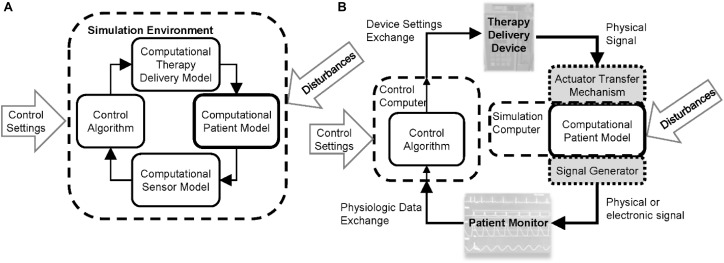
Computational test setups for physiological closed-loop controlled medical devices. **(A)** Fully computational testing uses computational models of the therapeutic delivery devices, sensors, and computational patient model. Initial conditions are set for the controller settings and patient, delivery device, and sensor models. Simulated disturbance profiles such as timing of injuries or other therapies are input to challenge the controller. The testing may be run within a single simulation environment. **(B)** Hardware-in-the-loop testing uses a computational patient model and one or more of the computational device models are replaced with the physical devices. This requires the use of actuator transfer mechanisms to convert therapy delivery device outputs to digital signals received by the computational physiological model and/or signal generators to convert the output of the computational patient model to signals that can be recorded by the patient monitor.

Physiological closed-loop control medical devices are life-sustaining safety-critical systems and establishing their safety requires characterizing the performance under inter- and intra-patient variability, verifying system implementation, assessing the system response during expected functional and clinical disturbances, and assessing the clinical usability (Parvinian et al., 2018). Evidence to demonstrate the performance and safety of any medical device, in general, can include a combination of data from bench, animal, clinical, and computational testing (Faris and Shuren, 2017). For PCLC devices, the latter might involve running computer simulations that use the devices or models of the devices to control a **computational model of patient physiology**. Closed-loop systems in a wide range of engineering fields have a long history in model-based design and evaluation, in which the control algorithm or the hardware device is tested using computational models of the system. In the design phase, this can allow the control designer to acquire insights on system dynamics and enable rapid device iterations, lending confidence to a control algorithm by conferring a sufficient level of performance and robustness against conceivable variabilities and challenging scenarios. Model-based evaluation approaches can be used to rigorously stress controllers in a wide range of simulated scenarios. The type of testing can be either using completely *in silico* approaches where computational models of the patient and device components are used or hardware-in-the-loop methods where a CPM is used with the actual medical devices, Figure 1. For PCLC devices, these approaches have the potential to demonstrate performance and complement clinical trials by evaluating the device under a broader range of conditions that might not occur or be possible to evaluate in a clinical setting. Therefore, computational testing with mathematical models of patient physiology could advance the development of novel PCLC medical devices, and positively impact patient care, as long as the evidence is sufficiently credible for performance assessment.

A challenge to performing and relying on computational testing of PCLC systems is that it requires a computational model of the patient’s physiology. The physiology of interest can be complex with, for example, significant non-linearities and time-varying components (Hester et al., 2011). The degree to which the computational results can be relied upon as evidence of the device performance will heavily depend on the **evidence supporting the credibility of the computational patient model to represent the relevant physiology**. Therefore, for computational testing of safety-critical PCLC medical devices, the evidence supporting the CPM is a critical component to interpret and generate credibility in the computational testing simulations. The need for validating physiological models in a systematic way to increase credibility in the results that they generate has long been recognized (Cobelli et al., 1984).

Establishing the credibility of a computational model involves gathering evidence from different activities, such as *verification and validation (V&V), calibration, identifiability and sensitivity analysis, uncertainty quantification, and applicability analysis* (see Table 1 for definitions). In the context of computational models, verification asks the question, “did you solve the equations correctly,” whereas validation asks, “did you solve the correct equations?” Validation involves comparing model results with real-world data. It is generally accepted that this data should be distinct to data used in model development, but otherwise the precise meaning of validation is subject to interpretation, both within communities (Roache, 2009) and across communities (Bellocchi et al., 2011; Pathmanathan and Gray, 2018). Calibration, or tuning, involves fitting parameters in the mathematical equations (i.e., model form or model structure), such that the output of the model has a “good” fit to experimental data.^1^ *Calibration* is a model development activity and not a model assessment activity; nevertheless, calibration often results demonstrating good agreement are regularly presented as evidence for credibility of physiological models. *Identifiability analysis* evaluates the reliability of the parameter estimation procedure and resulting parameters (Cobelli et al., 1984; Batzel et al., 2012). *Sensitivity analysis* assesses how sensitive the model output is to different values of the model inputs (Saltelli et al., 2008). *Uncertainty quantification* involves identifying the sources of uncertainty in the model, quantifying the uncertainty of the sources using probability distributions, and then propagating the uncertainty through the model to determine the impact on the output (Smith, 2013). *Applicability analysis* involves a systematic evaluation of the relevance of the validation evidence to support using the computational model for the specific proposed application of the model, or COU. This type of analysis can be done if validation evidence is already gathered or can be used for planning to gather evidence, and provides transparency on the assumptions and decisions made to use a model (Pathmanathan et al., 2017).

**Table 1:** Descriptions of terms important to establishing credibility of a computational model.

Term	Description
Context of use – COU	A statement that defines the specific role and scope of the computational model used to address a question of interest (ASME, 2018).^a^
Verification	The process of determining that a computational model accurately represents the underlying mathematical model and its solution (ASME, 2018).^b^
Validation	The process of determining the degree to which a model or a simulation is an accurate representation of the real world.
Sensitivity analysis	The process of determining how a change in a model input (e.g., parameters or initial conditions) affects model outputs.
Identifiability analysis	The process of determining the reliability of parameter estimates from model structure and experimental data.
Calibration	The process of tuning or optimizing parameters in a computational model to minimize the difference between model outputs and real world data.
Uncertainty quantification – UQ	The process of determining the uncertainty in model inputs (e.g., parameters or initial conditions) and computing the resultant uncertainty in model outputs (ASME, 2018).^c^
Applicability analysis	The process of assessing the relevance of the validation activities for a computational model to support the use of that model for a COU (ASME, 2018).^d^


*Additional comments are provided in the footnote. ^*a*^ The COU can also include the other sources of evidence that will support the question of interest. ^*b*^ Verification activities are outside the scope of our review. ^*c*^ Common reasons for uncertainty in model inputs are measurement error or inter-/intra-individual variability. ^*d*^ Detailed discussion in Pathmanathan et al. (2017). UQ usually involves quantifying such uncertainty using probability distributions.*

The model COU drives the rigor of and results from each activity that is necessary to establish credibility. For example, a CPM used in the early stages of designing a PCLC algorithm likely does not require the same rigor as a CPM used to determine the use population for the PCLC device. V&V-based processes for model assessment are well-established in traditional engineering disciplines, although they continue to evolve; however, the same rigorous attention has yet to be given to biomedical applications. Therefore, the American Society of Mechanical Engineers Verification and Validation Subcommittee, V&V 40 Verification and Validation in Computational Modeling of Medical Devices (ASME V&V 40) recently proposed a “risk-informed credibility assessment framework” to help determine the “amount of V&V” needed to support using a computational model for applications related to medical devices (Morrison et al., 2017; ASME, 2018). With this framework in mind, we present a review of the current uses of CPMs in the development of critical care PCLC devices and the evidence used to support the CPM for that purpose. We focus on three PCLC device areas intended for critical care medicine: hemodynamic stability, mechanical ventilation, and anesthetic delivery.

Our objective was to review the potential roles of CPMs to answer questions about PCLC system design and/or evaluation, and to identify the credibility evidence that supported the CPM for that purpose. The scope of this review included CPMs that were mathematical models of individual physiological systems, data-driven models, integrated physiological systems models, or PK/PD models. We considered studies with PCLC related to hemodynamic stability (primarily fluid resuscitation or vasoactive drug delivery), anesthetic delivery, and mechanical ventilation. Studies of animal or clinical testing of a PCLC system without any computational testing using a CPM were out of scope. Moreover, while models of the closed-loop system components (e.g., physiological monitors, infusion pumps) need to be considered for computational testing of PCLC systems, these models were also out of scope. Furthermore, it is important to distinguish between *validating the controller* and *validating the CPM; our reviewed focused solely on the latter*. We considered the following regarding the use of the CPM:

• the source of the model (whether designed for a specific COU in the article or taken from previous works or combination of two);
• information on the selection of parameter values including if parameters were taken from previous studies, averaged over population, calibrated during the model development process, or combination of these methods;
• whether sensitivity and/or identifiability analyses were performed;
• whether uncertainty quantification was performed;
• comparisons of model performance to experimental data used in model development/training/calibration processes (e.g., quality of fit)
• independent validation data, namely the comparisons of model performance to experimental data not used in any stage of model development (e.g., quality of predictive performance)
• for models previously reported, justification provided to support credibility for the current COU in the article;
• and, for models modified from previous reports, justification provided as to why any previous validation still supports the model use given the changes to the model.

Sections “Closed-Loop Systems for Hemodynamic Stability,” “Closed-Loop Systems for Mechanical Ventilation,” and “Closed-Loop Systems for Anesthetic Delivery” review the identified uses and validation approaches of CPMs in the development and evaluation of PCLC systems for hemodynamic stability, mechanical ventilation, and anesthetic delivery systems, respectively. Section “Discussion” discusses the results of the review, provides an appraisal of the state of model credibility assessment in the reviewed literature as a whole, and frames the content in relation to physiological model validation frameworks and the recent ASME V&V 40 framework.

## Closed-Loop Systems for Hemodynamic Stability

### Use of Computational Patient Models

Physiological closed-loop control controllers have been developed for resuscitation and vasoactive drug delivery systems with the objective of performing fluid resuscitation and/or hemodynamic stability. These devices monitor and control hemodynamic variables such as MAP or CO. Controllers used in hemodynamic stability systems adjust the infusion rate and/or time of delivery of fluids (e.g., colloids, crystalloids, or blood) and/or vasoactive drugs (e.g., SNP, phenylephrine). A variety of controller designs have been tested with CPMs including single input-single output adaptive and model predictive controllers (Malagutti et al., 2013; Silva et al., 2017), rule-based learning systems (Rinehart et al., 2011), PID controllers (Wassar et al., 2014; Bighamian et al., 2016), and multi-input–multi-output systems that control multiple drugs simultaneously (Held and Roy, 1995; Huang and Roy, 1998; Rao et al., 2000). System designs may include supervisory components that add a layer of safety by monitoring for known system limitations, such as noise/signal artifacts in the sensed physiological variables that could adversely impact the controller performance (artin et al., 1992).

Multiple studies have used CPMs to assess controller performance across a broad range of physiologic responses. This type of testing can help establish patient populations for which a new control algorithm may be safe for use by varying parameters within the CPM to represent different types of patients and expected responses. Kashihara et al. (2004) developed a model to compare the robustness of various control algorithms to the sensitivity of the MAP response to norepinephrine. The evidence from the simulation studies were used to initiate animal studies to further evaluate their controller designs. Bighamian et al. (2016) developed 100 different configurations of a CPM representative of hypovolemia to assess their PID controller for a fluid resuscitation system against a range of autonomic, cardiac, and hemodynamic conditions by varying gain parameters in their model. Similarly, Rinehart et al. (2013) assessed the robustness of their fluid resuscitation controller to varying patient weight and cardiac contractility. These types of studies enable an estimate of the potential distributions in controller performance metrics that may be expected and can be used to identify patient conditions that may result in unsafe controller performance.

Computational patient models are commonly used in the design and evaluation of closed-loop hemodynamic stability systems to assess controller performance and robustness to changing patient conditions over time. This allows an assessment of how the controller performs during intra-patient variability simulated by changing model parameters over time. A model of the MAP response to SNP designed to develop and test a controller to maintain MAP by titrating delivery of SNP (Slate, 1981) has been modified and further expanded on by multiple groups for this purpose. These modified versions of the model have been used to design and evaluate controllers capable of tracking and responding to a patient with a changing physiological state (Chen et al., 1997; Malagutti et al., 2013) and to address non-linear elements in the cardiovascular system (Polycarpou and Conway, 1998; Gao and Er, 2005). For example, Malagutti et al. (2013) considered wide variance ranges in parameters based on information in the clinical literature to enable their controller to respond to potentially unknown patient conditions. Wassar et al. (2014) designed a stochastic non-linear model of MAP response to phenylephrine that considered both inter and intra patient variability using data from swine experiments. Additional model components were added to model respiratory effects and disturbances such as hemorrhage or the presence of other vasoactive drugs. The model was used to design and investigate a controller under various simulated scenarios before testing the controller in swine experiments (Wassar et al., 2014). Luspay and Grigoriadis (2015) designed a Kalman filter to track time-varying parameters for continuous scheduling of a robust linear parameter-varying controller. A non-linear CPM was developed to test the controller during scenarios where patients could be sensitive, nominal, or insensitive to phenylephrine. This was done by drawing values for each parameter from pre-defined probability distributions (Luspay and Grigoriadis, 2015).

Held and Roy (1995) used a CPM of the canine cardiovascular system to design and assess a controller intended to adapt to patient SNP sensitivity and confirm that the controller maintained SNP and dopamine within specified limits. Rao et al. (2000) expanded the canine cardiovascular model to develop a multi-input-multi-output controller. This controller was intended for hemodynamic and anesthetic stabilization by modeling multiple hemodynamic measurements and a depth of anesthesia measure to control five vasoactive and anesthetic drugs. A linearized version of the model was used as part of their model predictive controller and a non-linear version able to simulate multiple pathological conditions was used to evaluate the controller performance as the simulated patient’s response changed over time due to the condition (Rao et al., 2000). Coté et al. (1995) modeled cerebral spinal fluid compartments to design and evaluate the stability and robustness of a closed-loop intracranial pressure regulation system against a range of relevant conditions.

Real-time implementations of CPMs enable more realistic testing of the complete system under expected functional challenges that might be exhibited during normal use. artin et al. (1992) developed a pulsatile cardiovascular systems model intended to test the PCLC system in real-time when faced with infusion rate limits, rapid physiological changes in MAP, and artifacts in the MAP signal. Woodruff et al. (1997) expanded this by combining it with PK/PD models for multiple vasoactive drugs. Moreover, they modeled the interactions between drugs and baroreceptor components to provide a generalized cardiovascular model for the design and evaluation of closed-loop hemodynamic devices (Woodruff et al., 1997). Silva et al. (2017) also enabled real-time testing of embedded controller designs by implementing a model on an embedded system.

An additional use of CPMs for PCLC systems is to be used as part of the control algorithm. For example, Urzua et al. (1999) demonstrated that using a simplified model of arterial pressure control within the arterial pressure controller proposed in Slate (1981) could improve the MAP response time to the therapy. To incorporate the numerous physiologic mechanisms involved in arterial pressure control into their system design, Nguyen et al. (2008) used a reconstructed version of Guyton’s model within their SNP control system (Urzua et al., 1999). Frei et al. (2000) used a model predictive controller to control MAP during surgical stimulation by titrating anesthetic agents. A separate model of the MAP response to stimulation was used to evaluate the model predictive controller.

### Credibility Evidence

The development of cardiovascular CPMs has a long robust history. Therefore, many CPMs used for development of PCLC systems for hemodynamic stability are modified versions of previously described models. When existing CPMs were modified, the impact of the modification on the model performance was rarely assessed or validated for the new COU. The model of MAP response to SNP initially developed by Slate (1981) was used and modified by numerous groups to evaluate controller designs during periods of disturbances and time-variance in patient responses as well as handing of parametric uncertainty related to patient responses. Multiple papers (Polycarpou and Conway, 1998; Gao and Er, 2005) modified the Slate model to assess their controller performance against drug sensitivity by modifying parameters to establish the safety of the controller given unknown patient conditions. However, additional credibility evidence was rarely provided to support that the model output remained physiologically relevant across the range of modified parameters.

Qualitative assessment of steady-state and dynamic responses was commonly presented to support model use. Rinehart et al. (2013) describe the simplicity and previous validation to support using a Frank-Starling baroreceptor model (Upton R.N. and Ludbrook G., 2005; Upton R.N. and Ludbrook G.L., 2005) to assess their closed-loop fluid resuscitation system. The model CO response to blood loss was presented to demonstrate that their modifications produced a physiologically plausible response (Rinehart et al., 2013). While acknowledging the limitations of the model, the authors discuss the suitability for the questions being addressed in the study. Bighamian et al. (2016) used a previously developed model (Ursino, 1998; Ursino and Magosso, 2003) to simulate 100 different hypovolemic patient conditions. An assessment of the distributions of hemodynamic variables was made to consider if the hemodynamic responses generated by the different parameter configurations were reasonable. The author noted that the model did not include key physiological elements such as urine excretion that may impact the fluid volume and thus the model results. Urzua et al. (1999) provided a detailed description of the physiological basis for their model used within their controller. The qualitative behavior of the model in the presence of disturbances was contrasted against experimental data to demonstrate the physiologic relevance and identify behaviors that may not be physiologic.

Woodruff et al. (1997) provide a comprehensive description of the development and rationale behind the various components of their cardiovascular simulator that allowed inputs for multiple vasoactive drugs and presented qualitative and quantitative assessments of model performance. A sub-model approach to validation was used where additional components were subsequently added to the model and then model predictions were qualitatively and quantitatively compared to published animal and clinical data (Woodruff et al., 1997). An assessment of the realism of the model was also performed by having anesthesiologists with varying levels of experience control SNP using a simulator.

A series of articles built on the canine cardiovascular model developed and evaluated in Yu et al. (1990). Held and Roy (1995) provided a qualitative assessment of the dynamic and steady-state behavior of the model under specific conditions with a discussion of how this relates to expected physiological changes following their modifications to improve the run-time and agreement with experimental results. Rao et al. (2000) added of a PK/PD model for propofol to use the model for testing simultaneous hemodynamic and anesthetic control systems. The authors note that they varied circulatory parameters to ensure that MAP and heart rate responses matched experimental observations, and propofol model parameters were then tuned so that the model matched steady-state results (Rao et al., 2000). The results of the calibration procedure were presented along with simulation results to assess the model predicted hemodynamic responses due to increasing propofol infusion.

Kashihara et al. (2004) developed a data-driven model of the MAP response to norepinephrine from experimental data tracking MAP during infusion of norepinephrine. Data from three experiments were used to fit the model, and a comparison of the experimental and simulated responses from a single animal was presented to support the model performance. Nguyen et al. (2008) also used data from animal experiments to tune parameters in an SNP infusion model that was connected with a larger cardiovascular systems model. Qualitative assessment was presented that compared the blood pressure response to SNP on the calibration data used to tune the SNP infusion model.

Wassar et al. (2014) used data from swine experiments to develop a stochastic model of MAP response to phenylephrine that enabled varying the sensitivity of MAP to the infusion rate of phenylephrine. An initial portion of experimental data was used to identify model parameters and then those parameters were applied to the model and predict the remaining portion of the experimental data. Results were presented from at least one animal used in development of the model that showed the measured and predicted responses; the authors noted that similar results occurred for the other animals. The model was determined to be appropriate by the authors because it included saturation effects and allowed for inter and intra subject variability to be assessed.

Frei et al. (1997, 2000) evaluated the trade-off between model performance and model order when considering the appropriate model to use for testing the response of their MAP controller to anesthesia and disturbances. In the manuscript, they note the challenges with using high order physiological models and present evidence of their model performance on a single case of experimental data.

## Closed-Loop Systems for Mechanical Ventilation

### Use of Computational Patient Models

The controllers employed in closed-loop mechanical ventilation devices adjust ventilation parameters to maintain blood gas levels or other respiratory-related variables within a physiologic range of interest. Computational modeling of the cardiorespiratory system plays a key role in design (Martinoni et al., 2004; Jiang et al., 2016; Pomprapa et al., 2017) and evaluation (Morozoff and Saif, 2008) of closed-loop mechanical ventilation algorithms. The majority of efforts behind the closed-loop design and evaluation of a mechanical ventilation device is to continuously adjust blood oxygenation levels [i.e., SpO_2_ (Tehrani, 1993, 2012; Iobbi et al., 2007; Morozoff and Saif, 2008) and SaO_2_ (Yu et al., 1987; Pomprapa et al., 2017)], carbon dioxide levels [end-tidal CO_2_ fraction (Martinoni et al., 2004; Hahn et al., 2012; Kim et al., 2016)] or respiratory variables including respiration rate and tidal volume (Flechelles et al., 2013). Adjustments are made by varying the level of therapeutic settings on the device including fraction of inspired oxygen (FiO_2_) (Morozoff and Saif, 2008; Pomprapa et al., 2017), tidal volume (Martinoni et al., 2004), and positive end expiratory pressure (Tehrani, 2012; Flechelles et al., 2013). A wide range of controller types have been applied including model predictive controllers (Fernando et al., 1995; Pomprapa et al., 2017), PID and fuzzy controller (Morozoff and Saif, 2008), robust controllers (Sano et al., 1988; Pomprapa et al., 2013), and rule-based expert systems (Fernando et al., 1995).

Yu et al. (1987) and Sano et al. (1988) both developed transfer functions to relate blood oxygen levels to FiO_2_ by linearizing the gas transport model in order to simplify the controller design process. These models were used to design controllers to adjust PaO_2_ by titrating FiO_2_. Sano et al. (1988) further developed a linearized model relating rate of breathing to blood CO_2_ levels.

Many CPMs used in the design and evaluation of PCLC mechanical ventilation devices were developed from foundational studies in modeling of cardiorespiratory system such as Fincham and Tehrani (1983); Rideout (1991) and Morozoff et al. (1993) and then with improvements from Tehrani (1993) and Morozoff and Saif (2008). Fincham and Tehrani (1983) improved on past models of respiratory systems by accounting for physiological processes such as respiratory work output and internal body feedback systems. This model forms the basis for design and evaluation of closed-loop oxygenation devices in adults (Tehrani, 2012). The performance of the control system was evaluated in simulation experiments representing different physiological conditions including low respiratory compliance and hypoventilation. The model presented in Fincham and Tehrani (1983) involved mass balance dynamics of gas transport and exchange, metabolism dynamics, chemoreceptors, and endogenous respiratory control mechanisms. The original model was modified by adding characteristics of the neonatal respiratory system such as lung shunts to enable design of controllers intended for neonatal oxygenation control (Tehrani, 1993). Morozoff et al. (1993) and Morozoff and Saif (2008) developed a model that consisted of submodels representing the cardiovascular system and the respiratory system including the effect of shunts. They first developed a model for adult cardiorespiratory system and then used an allomeric approach and proportional scaling to derive model parameters for neonatal applications. They evaluated fuzzy logic and PID controllers by using this model to simulate various neonatal conditions including changing oxygen affinity, desaturation pulse duration, patient motion conditions, and combinations of these.

Data-driven models of EtCO_2_ have been presented for controller development (Hahn et al., 2012; Kim et al., 2016). Kim et al. (2016) described lowering the order of compartmental models to capture CO_2_ transport dynamics with and without CO_2_ transport delay that could be used to design and test controllers. Hahn et al. (2012) developed an empirical low order model relating EtCO2 to minute ventilation to design a PI compensator using clinical data to identify the model parameters.

Pomprapa et al. (2017) evaluated a Policy Iteration Algorithm (PIA) controller based on a model of blood oxygenation. The model was used to evaluate the performance of various iterations of the PIA controller in terms of settling time. The parameters of the model were identified by changing FiO_2_ settings and inducing acute respiratory distress syndrome in a large animal experiment. For further implementation of control system design, they approximated the non-linear identified model with a simplified linearized model. Iobbi et al. (2007) reported a model of oxygen transport to evaluate a controller intended to adjust FiO_2_ based on pulse oximetry feedback. They assumed second order transfer functions with delays to relate blood oxygen saturation levels to inspired oxygen. The model was used to simulate hypoxic events from artificial disturbances representing variable fluctuations in patient pathophysiology.

### Credibility Evidence

Yu et al. (1987) provided model formulation and descriptive information of the physiological process behind blood oxygenation, relying on their previous work to justify the structure of a first order transfer function relating arterial partial pressure (PaO_2_) to FiO_2_. They selected ranges for model parameters but did not provide any information about the validation of these ranges or the sources they were derived from. Similarly, Sano et al. (1988) designed a model of both O_2_ and CO_2_ gas transport for robust controller design. Their rationale for constructing a new model was that the previously developed cardiorespiratory models were too complex for control system design.

The foundational studies presented in Fincham and Tehrani (1983) and Morozoff and Saif (2008) generally provided great details of model formulation and structure. The modeling approach and rationale for inclusion of model components, for example dynamics and chemical control of gas levels in blood, were provided. In Fincham and Tehrani (1983) model evaluation involved comparing the model performance at various conditions including hypoxic, hypercapnic, and exercise conditions. Direct comparison with experimental results reported in literature were performed only for resting and hypercapnic conditions. Specifically, the comparison included numerical values of selected model outputs (e.g., arterio-venous gas difference) and not of the entire range of variables that would be utilized in future studies involving design and evaluation of closed-loop mechanical ventilation devices such as Tehrani (2012).

Morozoff et al. (1993) described a model with simplifying assumptions such as not accounting for the removal of oxygen as CO_2_ during expiration in the mass balance of O_2_. A more detailed compartmental model of the respiratory and cardiovascular systems was described in Morozoff and Saif (2008). The cardiovascular system model was compared with the model presented in Rideout (1991), although the level of agreement and specific conditions were not specified. The study provided a clear list of assumptions for the cardiovascular model such as uni-directionality and non-pulsatile blood flow and perfect mixing of blood. The authors used proportional scaling and parameter values of mammals similar in weight and surface area to neonates to select model parameters specific for neonatal patients circulatory and respiratory systems. The authors state that the model was validated in stages first using Rideout data (Rideout, 1991) for the adult cardiovascular system and then using published data for the neonatal model. For neonates, the model was compared qualitatively with physiology text books.

Martinoni et al. (2004) provided a simplified model of that presented by Chiari et al. (1997) where they assumed constant CO and constant oxygen saturation in both arterial and venous blood. The model was “considered sufficiently descriptive for closed-loop control purposes” (Chiari et al., 1997).

Iobbi et al. (2007) used a model of the oxygen dissociation curve proposed by Severinghaus (1979). They assumed that the oxygen response is a function of three factors: a baseline oxygen partial pressure, a driving partial pressure which was modeled as a sinusoidal function, and a patient oxygen flow dependent term which was modeled as a second order differential equation whose parameters were selected from literature. The authors acknowledged that the parameters selected are based on average values and do not represent patient variability.

Pomprapa et al. (2013) described the input–output relationship of minute ventilation and EtCo_2_ and compare a first order linear model and a non-linear model. The authors used root-mean-square-error (RMSE) of an estimated dataset and a “validated” dataset to compare the model results. They also provided qualitative comparison of linear and non-linear models. Model parameter values were calibrated by collecting experimental data from a single healthy human subject with homogenous lungs and normal body mass index. The authors suggested that the model was validated using the single human subject data by mentioning comparison of the estimated model “with a validated dataset,” although it is unclear from the manuscript how this dataset was developed and whether the validation dataset involved measurement of model output independent of model calibration.

Hahn et al. (2012) developed an empirical model with coefficients identified from data. The authors demonstrate the similarity between their affine model relating minute ventilation to EtCO_2_ concentration to pharmacological modeling. They use this analogy to provide physiological interpretation of the model as having two compartments representing body and lungs. Respiratory data of 18 pediatric patients from an anonymized repository were used for model calibration. Model fit was quantitatively compared with experimental data set using RMSE and coefficient of determination. Kim et al. (2016) identified model parameters from clinical data. They demonstrated that inclusion of transport delay resulted in model prediction improvement compared with the same model without transport delay. Unlike the model with transport delay, the parameter estimation on the model without transport delay resulted in parameter values that are not physiologically plausible.

## Closed-Loop Systems for Anesthetic Delivery

### Use of Computational Patient Models

Computational physiological models have been widely used to design and evaluate closed-loop anesthetic delivery systems. The systems control level of consciousness (Elkfafi et al., 1998; Bibian et al., 2006a,b; Dumont et al., 2009; Hahn et al., 2012; Kharisov et al., 2012; Merigo et al., 2017), analgesia (Gentilini et al., 2002; Ionescu et al., 2014), neuromuscular blockade (Zhusubaliyev et al., 2014; Almeida et al., 2017) or combinations of these goals (Fang et al., 2014; Silva et al., 2014; Jin et al., 2018). Additionally a series of studies designed closed-loop propofol delivery with the specific aim of inducing and maintaining pharmacologic burst suppression based on processed EEG signal (Liberman et al., 2013; Westover et al., 2015). PCLC devices intended for control of hypnosis delivered propofol and used feedback variables such as bispectral index (BIS) (Kharisov et al., 2012; Merigo et al., 2017), WAVcns (Dumont et al., 2009; Hahn et al., 2012), or auditory evoked response (Elkfafi et al., 1998) to titrate anesthetic agents including propofol and isoflurane. Some studies combined the closed-loop delivery of hypnosis with analgesia leading to closed-loop co-administration of propofol and remifentanil (Ionescu et al., 2014; Jin et al., 2018). Neuromuscular blockade agents such as rocuronium and atracurium were used and controlled using feedback variables based on muscle movement (Silva et al., 2015; Almeida et al., 2017) and in some studies combined with closed-loop hypnosis delivery (Linkens and Mahfouf, 1992; Fang et al., 2014; Silva et al., 2014). Gentilini et al. (2002) used hemodynamic variables such as MAP and plasma concentration to titrate drug delivery.

Most CPMs used for design and evaluation of closed-loop anesthesia delivery systems have been derived using existing PK/PD models for the anesthetic drug of interest. Fang et al. (2014) constructed a library of widely used virtual subject models after investigating and collecting numbers of reported patient models for propofol, isoflurane, remifentanil, and atracurium administration. They combined the model database with a control algorithm database including PID control and model predictive control while facilitating four control modes (manual, automatic, switching from manual to automatic, and switching from automatic to manual). This created an anesthesia simulation platform imitating the clinical situation. A patient response PK/PD model of hypnotic drug isoflurane was adopted in Sreenivas et al. (2009) to generate, by varying the PK/PD parameters, 16 representative patients selected to cover the range of observed sensitivity from 972 patients. These 16 virtual patients were then used to evaluate and compare performance, including robustness and stability for expected surgical disturbances across six different controllers designed for hypnosis regulation, using BIS as the controlled variable. Liu and Northrop (1990) modeled patient perceived pain using a PK model of fentanyl, a triexponential weight function, and a steady state biochemical kinetic model. This was to represent the relationship between brain tissue drug level and CNS unbound opioid receptor ratio. They also used a relaxation pulse frequency model to convert pain to button pressing frequency (Liu and Northrop, 1990). This model was then used to assess performance of four controller algorithms in the presence of simulated patients with minimum, average, and maximum pain sensitivity. Alongside real world data Méndez et al. (2016) used a simulation to assess a fuzzy logic controller to regulate BIS using propofol. Simulation of patient response was conducted using a PK/PD model after choosing parameters to match real data from patients undergoing similar surgery procedures and with the same drugs as those used in the control experiments. Struys et al. (2004) developed a simulation methodology to compare performance between multiple controllers to control BIS using propofol. Virtual patients were generated using a computational model which incorporated PK/PD modeling along with effect relations modeling.

Nogueira et al. (2017) studied the performance of the controller proposed in Nogueira et al. (2014) to regulate BIS using propofol and remifentanil under the presence of model parameter uncertainties and introduced a retuning strategy for the model to recalibrate itself. Six simulated patients were developed by setting parameters of the model based on the data of real patient’s subjected to general anesthesia under propofol and remifentanil manually controlled by an anesthetist. Dumont et al. (2009) used PK/PD model results of a number of patients from different groups to choose controller parameters to design robust PID controllers and robust controllers based on fractional calculus to regulate hypnotic state of anesthesia using intravenous administration of propofol. Gentilini et al. (2002) used a PK-PD model as a part of their controller to control MAP by controlling analgesics. Puebla (2005) used a model (Gentilini et al., 2001) to design a cascade controller to control end-tidal anesthetic agent concentration in response to BIS sensor.

Mahfouf et al. (2003) published a series of studies using a multivariable model of anesthesia that combined various models obtained from classical PK/PD models, published literature, and the authors own identification experiments (Linkens and Mahfouf, 1992). These experiments involved collecting drug infusion rates (inputs) and MAP (outputs), although it is unclear if this was done on measured or simulated data. They used the models to synthesize a model predictive controller that would enable the next control action to be determined based on the output of the models. Méndez et al. (2016) developed a fuzzy model predictive controller based on fuzzy modeling of the patient PK/PD response.

Westover et al. (2015) used Schinder’s PK/PD models of propofol (Schnider et al., 1999; Schnider and Minto, 2008) to construct a robust PID controller for maintenance of burst suppression. The authors used an identification procedure to determine subject-specific PD parameters in a rodent study. PK variability was determined using published estimates of the coefficient of variation in PK parameters. The approach was similar to a series of studies in Bibian et al. (2006a,b), Dumont et al. (2009), and Hahn et al. (2012) on closed-loop control of hypnosis using propofol that leveraged classical PK models of Schüttler and Ihmsen (2000) and classical modeling of PD using the Hill equation. The authors used such models to build controllers robust to parametric variability and disturbances. Minto et al. (2000) and Jin et al. (2018) continued the work done by Hahn et al. (2012) and designed a coordination controller that recursively adjusts the reference targets based on the estimated dose–response relationship of a patient using a classical PK/PD model of propofol and remifentanil.

Silva et al. (2014, 2015) introduced a Wiener model consisting of linear dynamics and a static non-linearity to characterize the response (train-of-four – TOF) to neuromuscular blockade drugs. The studies focused on developing minimal model for the purpose of controller design. Almeida et al. (2017) used Silva’s model presented in Silva et al. (2015) to design an adaptive controller to titrate rocuronium and control muscle movement, and Nogueira et al. (2017) used it to design a non-linear controller for controlling depth of hypnosis by titrating propofol.

Kharisov et al. (2012, 2015) designed a model for the purpose of designing a controller for automatic delivery of isoflurane based on BIS. The model was developed using clinical trial data collected in earlier studies (Lin et al., 2004; Beck et al., 2007; Kramer et al., 2008). In Kharisov et al. (2012) a black-box model was developed for the purpose of controller design, whereas in Kharisov et al. (2015) the same clinical data were used to estimate parameters of a gray-box linear time-invariant model structure for controller design. Subspace identification methods were used to construct models for six patients for the black box model while for gray box modeling, explicit parameters representing mean biases and an adaptive gain parameter capturing the time-varying nature of patient tolerance to the anesthetic agents were formulated.

### Credibility Evidence

Numerous studies relied on established PK/PD relationships previously reported for the drug of interest. Gentilini et al. (2002) adopted a PK model with parameters from literature and approximated the PD model with parameters based on anesthesiologists knowledge and experience. A linear relationship between MAP and effect site concentration was assumed, and the gain for the PD relationship was determined based on expert opinion. Qualitative and descriptive information of the model were provided. Linkens and Mahfouf (1992) leveraged PK/PD models from published literature combined with physiological models of blood pressure. Parameters for the Atracurium PK/PD model were taken from literature and measurements in the operating theater. The hypnotic effect of isoflurane was identified using published clinical data (Millard et al., 1988a,b). Descriptive information for structure and formulation of submodels (e.g., the isoflurane to muscle relaxation interaction model) were limited. Mahfouf et al. (2003) used a circulatory and inhalational anesthesia model structure previously reported in literature with a blood pressure delay parameter from their own previous work. They also combined the physiology model with a model of anesthetic phase change (Mahfouf et al., 2003). Unknown model parameters were identified using 400 points from isoflurane delivery rate (input) and MAP (output) data with a fit quantitatively evaluated using RMSE.

Liu and Northrop (1990) provided detailed description of the model and source of calibration data. Model parameters were fit so that the model output follows actual patient responses reported in previous work. Struys used the Schnider PK model and the PD model developed previously in their clinical work (Gentilini et al., 2002; Struys et al., 2004). Ten virtual patients were selected to represent different pharmacodynamic profiles defined by drug effect site concentration and effect relation combined with a delay parameter. The authors relied on their previous work and published articles of Schnider to create the virtual data (Gentilini et al., 2002). Similarly, Ionescu et al. (2014) designed a model predictive controller using established PK/PD models from Schnider and Minto (2008) along with their clinical experience to obtain model parameters.

Merigo et al. (2017) used the PK model with parameters from Gentilini et al. (2002) held at nominal values. They identified the PD parameters for the standard Hill equation. Elkfafi et al. (1998) provide qualitative description of how the model was designed to show the rate of change of depth of anesthesia with respect to propofol infusion rate.

A number of studies (Bibian et al., 2006a,b; Dumont et al., 2009; Hahn et al., 2012; Westover et al., 2015) have reported the design and evaluation of robust controllers by considering the variability associated with patient parameters for PK/PD models reported in literature. The structure and form of the compartmental PK and effect site PD models used in these studies were reported. The range of PK model parameters were obtained from published evidence while the PD model parameter ranges were identified using clinical data in Bibian et al. (2006a,b), Dumont et al. (2009), and Hahn et al. (2012) and animal data in Westover et al. (2015). Credibility evidence included references to previous studies.

Unlike previous studies that adopted classical PK/PD models, Méndez et al. (2016) developed a model of propofol impact on BIS using fuzzy modeling. Information on the form of the fuzzy model and rules was provided. To develop the model, experimental data from a clinical study that captured input (propofol) and output (BIS) measurements was divided into two training (15 patients) and validation (10 patients) sets. The fit of the fuzzy model was reported as compared with compartmental model for one patient, but it is unclear if this case represents a calibration scenario or is from a separate independent validation data set. Méndez et al. (2016) acknowledged that, because of actual population variability and presence of disturbances during surgery that may not have been captured in the model, the controller performance in operating room could vary with respect to the simulated results.

Silva et al. (2014, 2015) provided studies of identification of NMB and hypnosis. Weiner models to be used for closed-loop system design. The main emphasis of these studies was to develop parsimonious models by lowering the model order to make it amenable to controller design. Such models were subsequently adopted in Teixeira et al. (2014); Almeida et al. (2017), and Nogueira et al. (2017) for the purpose of controller design. Original modeling studies by Da Silva et al. (2012) mentioned validity of the previous model and note that the current model needs to be modified for the current COU. They reduced the model order by lumping the parameter values into a single parameter.

Kharisov et al. (2015) provided a description of their model and its formulation. Model parameters were estimated by calibrating the model to previously conducted clinical data. Portions of the patient’s data were selected during isoflurane infusion to identify model parameters. The model was evaluated using the same clinical trial data, and the output of the model was reported for one patient. The model did not appear to follow the experimental data beyond the portions in which calibration was performed. It appears that the segmentation and selection of intervals for parameter identification was based on the COU of the model which was to be used for BIS levels below 70. The model predictive capability was not evaluated on independent data that did not take part in parameter estimation. In Kharisov et al. (2012), the authors provided a description of the model and formulation. The fit of the calibrated model was quantitatively evaluated on the same clinical data and the authors determined that the fit achieved was “acceptable” for their COU.

## Discussion

By harnessing the power of model-based design and evaluation, it is possible to perform rigorous stress testing of PCLC controllers to study the system behavior under challenging functional and clinical scenarios. Advantages and benefits of model-based design and evaluation will become more pronounced as system complexity continues to grow. The future of automated critical care may include not only closed-loop control of a single physiological variable but simultaneous control of multiple physiological variables from independent devices (Bracco, 2011). These controllers will need to work in tandem to maintain the patient in a stable physiologic condition. There is a possibility, however, that the controllers may adversely affect one other, potentially creating hazardous conditions for the patient. Model-based design and evaluation has the versatility to quantitatively and systematically (i.e., in a modular approach) assess the potential impact of each controller on patient physiology and elucidate and properly mitigate potential risky conditions in which the controller(s) may fail or malfunction. However, the increased complexity in the control systems will require increasingly complex and sufficiently accurate patient models, and thus mechanisms to assess model credibility will need to be more rigorously employed. We identified several uses for CPMs in the design and evaluation of PCLC systems ranging from systematic design of controllers and control law, as part of the controller (e.g., model predictive control), evaluating controller performance to different patient responses, evaluating controller performance to changing responses over time, and integrating with the PCLC physical system to assess real-time performance and implementation of safety supervisory modes. We further provide a review of the credibility evidence provided to support a broad range of those uses and will discuss the ASME V&V 40 framework.

### Modeling for Systematic and Iterative Design

Computational patient models used for design purposes may be termed “control oriented” or “control relevant” models characterized by low order, parsimonious in parameters, and linearizable models to enhance controllability and observability (Silva et al., 2015, 2017; Kim et al., 2016). They are generally used to synthesize a controller around an operating point and may not be applicable to a broad range of clinical (e.g., patient variability) and functional (e.g., noise disturbance) uncertainties. With this COU, CPMs are used with varying degrees of influence on controller function. For example, CPMs may be used for tuning PID controller gains (Westover et al., 2015), to inform the next action and set point of a controller (e.g., adaptive and model predictive controllers) (Malagutti et al., 2013) or to synthesize controllers robust against uncertain conditions (Dumont et al., 2009; Hahn et al., 2012). Establishing the credibility of the CPMs for such uses may include evaluating qualitative (e.g., model order, simplicity, transparency) and quantitative (e.g., linearizability, identifiability, predictive accuracy) characteristics. However, the rigor of these activities may vary. For example, a model predictive controller may need more rigor in validation evidence as compared to a conventional PID controller due to influence of the model on controller output.

### Modeling for Evaluation of Controller Designs

Although controllers are constantly evaluated in an iterative manner to enhance design, CPMs may also be used for final evaluation of controllers to inform risk analysis. Such evaluation may be conducted either completely *in silico* (Kovatchev et al., 2009; Parvinian et al., 2018) or using a physical system integrated with the computational model (Parvinian et al., 2018) (Figure 1). This COU for the CPM requires them to represent a broad range of clinical and functional scenarios to be able to stress-test the controllers. The CPMs used for this context generally are more complex (i.e., have higher order and more parameters) compared to models used for systematic design of controllers. The credibility-related requirements for this COU overlap to some degree with those used for the design. For example, in both cases the models are expected to have identifiable parameters and model predictive accuracy. However, in the case of models used for final evaluation the rigor for each credibility factor may be higher due to the influence of the model on decision and decision consequences.

Few of the studies in both engineering and physiology fields included in this review have rigorously tackled the fundamental question of what makes a model sufficiently credible for a specific task in the design and evaluation of PCLC systems. With regards to evidence needed to assess credibility, we found a broad spectrum in the type and rigor of credibility evidence presented for CPMs, but there was no clear link between how the CPMs were used and the rigor and extent of the credibility evidence. The evidence ranged from mostly qualitative descriptive information and in some cases quantitative assessment of the model performance, although it is not always clear if this was performed on independent data or calibration data. The authors usually did not report on how the decision was made about the type and rigor of the credibility evidence needed to support the CPM and typically the details of such evidence were limited. Our review of CPMs for the COU of designing controllers indicates that most studies focus on developing and using simplified models through lumping of parameters and model order reduction in order to enhance feasibility of controller synthesis. In other words, most studies focused on whether a controller *can* be designed and not whether it *should* be designed using the investigated CPM. The degree to which quantitative credibility-related requirements such as predictive accuracy were considered is unclear for various applications in anesthesia, mechanical ventilation, and hemodynamic stability articles reviewed.

While most of the studies presented some qualitative assessment of model output, the application of more formal assessment techniques that provide systematic and quantitative information about the model performance, such as sensitivity analyses, uncertainty quantification, and comparisons to independent experimental data to assess the predictive capability of the model was limited.

### Role of Uncertainty Quantification in PCLC Design and Evaluation

A significant model attribute affecting credibility of models used for both systematic design and evaluation of PCLC controllers is the ability of the model to capture interpatient and intra patient variability as well as variability in disturbance scenarios experienced by critical care patients. This variability inherently establishes merit for using a closed loop system to account for the effect of variability and disturbances on the system output and transfer it to the manipulated variable. Adequate characterization and quantification of uncertainty for an application can therefore result in much more robust controller design and more meaningful controller evaluation methods. Recently some of these challenges related to uncertainty in patient and system response to disturbances have formed the impetus for synthesizing PCLC controllers based on modern control synthesis methods based on robust control theory (Bibian et al., 2006a,b; Dumont et al., 2009). While this type of controller presents a powerful tool with the potential to guarantee that control specifications such as stability and robustness are met, the models used in such methods need to produce clinically relevant and widely variable disturbance scenarios. Furthermore, uncertainty due to oversimplifying assumptions embedded in a CPM need to be considered. For robust control applications (Dumont et al., 2009; Westover et al., 2015), overall model uncertainty and the uncertainty bounds must be quantified and validated for model based design and evaluation. In medicine, such bounds will be a function of the patient population, patient characteristics, type of procedure, amount of drug infused, drug co-administration, and concomitant therapies.

Another method of handling inter-patient/intra-patient variability has been to design model predictive controllers (Mahfouf et al., 2003; Méndez et al., 2016). In this context, the model used for design of controllers is not static (as is the case with robust control). The model directly affects the outcome of the controller as it is part of it. For example, in model predictive control of anesthesia, a forecast of the output is communicated to the controller based on a mathematical model (Mahfouf et al., 2003). As such the credibility evidence needed to establish validation and uncertainty quantification of such models for this COU may require greater rigor due to the consequence of model output on the control function.

The issue of modeling uncertainty in anesthesia and critical care environment specifically is further complicated by multi-endpoint nature of therapy. For example, delivery of anesthesia is inherently a multiple-input-multiple-output system consisting of hypnosis, analgesia, and NMB (Beck, 2015). Most PK/PD models are designed for single input system and do not account for potential drug interactions. Furthermore PK/PD models are empirical and lack mechanistic insight into the physiological systems affecting the response. For example the pharmacodynamic parameter that represents sigmodicity of the effect does not have physiological relevance (Beck, 2015). This may complicate parameter uncertainty quantification particularly for designing controllers to maintain and adjust physiological endpoints such as respiratory rate, cardiac output, and mean arterial pressure. This challenge is likely to surface when PCLC devices intended for anesthesia are designed to work in tandem with, for example, PCLC mechanical ventilation and hemodynamic stability devices.

### Previous Work on Computational Physiological Model Validation

There are a number of contributors who helped lay the foundation for validation of physiological models. Cobelli et al. (1984) introduced a validation process for simple and complex physiological models. Simple models were regarded as those with plausibility of performing classical identification procedures whereas complex models were those with numerous parameters that made system identification and parameter estimation not possible. Their work outlines fundamental concepts to address credibility of models. They introduced concepts of model internal consistency, algorithmic validity, and external validity concepts such as empirical, theoretical, heuristics, and pragmatic validity. They also provide a step by step procedure for testing validity of simple models by first examining identifiability properties, followed by the goodness of fit, analysis of residual errors, and, finally, overall plausibility of model attributes (e.g., parameter, structure, behavior). For complex (and simple) models with unidentifiable parameters and for models with a small number of variables accessible for direct measurement, they recommend first employing model simplification, decomposition of a complex model into simpler sub-models, and improving experimental design/testability of the model, followed by performing adaptive fitting and finally checking for model plausibility. Batzel et al. (2009) provided steps for model validation including classical and generalized sensitivity analysis, subset selection, evaluation of local characteristic of parameter estimation process, experimental design and data quality checks, and global analysis. Summers et al. (2009) also recognized that whenever possible, model outputs should be compared with experimental outcomes for more consequential conclusions about model validity. They summarized three criteria for supporting the validation of large-scale integrated physiological models including a qualitative assessment of changes in the model output and a quantitative assessment of the steady-state and dynamic response of the model, while noting that these activities should be performed considering the clinical COU of the model (Summers et al., 2009; Hester et al., 2011). Some recent articles proposed systematic assessment methods relevant to physiological models and these two in particular discuss the importance of identifying and, when possible, quantifying uncertainty (Friedrich, 2016; Patterson and Whelan, 2017). While these frameworks present different methods for performing validation and assessing model performance, they do not present a method for determining the level of rigor or the extent of the evidence needed to determine that the model is sufficiently credible for a specific COU. Therefore, to complement the foundation studies, we present an overview of a new consensus standard from the subcommittee ASME V&V 40. The standard presents a risk-informed credibility assessment framework developed by a broad range of stakeholders in the medical device industry (ASME, 2018).

### ASME V&V 40 Framework

**FIGURE 2:**
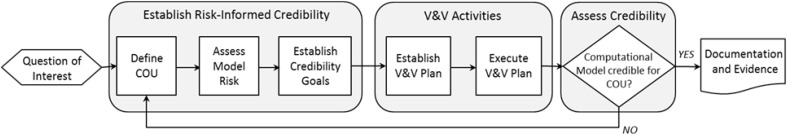
Process diagram of the ASME V&V 40 risk-informed credibility assessment framework. Modified from Morrison et al. (2017).

The framework presented in the ASME V&V 40 Standard builds upon established V&V methodologies and does not present a step-by-step guide on how to perform V&V; rather it guides the user (see Figure 2) to determine the necessary rigor of the V&V activities to deem the model credible for a COU. Thus, the framework begins with the user defining the question(s) of interest, which includes identifying a decision to be made, and the COU, a statement that describes the specific role and scope of the computational model. The level of rigor of the V&V is driven by model risk, the possibility that using the computational model to inform a decision can result in undesirable effects, such as patient harm. The main tenant of the V&V 40 framework is that the level of credibility required is commensurate with model risk. Model risk is determined by assessing both the *influence of the model* on the question of interest as compared to the other available evidence, and the *decision consequence*, which is the potential outcome of an incorrect decision. It is important for the user of the framework to carefully consider these two aspects of model risk, because once model risk is established for a COU it is used to drive the selection of credibility goals for the activities to be performed to establish model credibility.

The risk-informed credibility assessment framework provides a means to determine the level of credibility needed, and the associated activities, to support the computational model for a COU. The mechanism that drives credibility determination is 13 ‘credibility factors,’ which define different steps in the V&V process. As discussed above, the general V&V process includes verification, validation and applicability analysis. These are well defined in other documents, such as the NASA standard for model credibility assessment (Administration NAAS, 2013) and a Sandia National Laboratories report (Oberkampf et al., 2007). The V&V 40 framework breaks down verification, validation, and applicability analysis into more discrete steps. For example, in the process of validation, it is critical to examine the details of the experimental comparator as well as the computational model. Therefore, the credibility factors for validation include: “model form” and “model inputs” for the computational model, and “test samples” and “test conditions” for the experimental comparator. For the computational model, the credibility factors assess the rigor of the sensitivity analysis and uncertainty quantification performed and for which parameters these should be performed. With respect to the experimental comparator, which can be data from bench, animal, or clinical experiments, the two credibility factors assess the samples on which the experiments are performed, the quantity of samples, the conditions under which the experiments are performed, and the variety and variability of those experiments, along with the assessment and quantification of experimental uncertainty. Furthermore, for validation is a credibility factor that involves the assessment of the nature and types of comparison between the outputs from the computational model and the experimental comparator (i.e., qualitative or quantitative), along with the level of agreement between computational model and experimental data. The level of agreement is an outcome from the comparison; whether the level of agreement is sufficient is linked to the requirements for the COU.

Finally, there are two credibility factors related to applicability analysis, the relevance of the validation activities to the COU, and the relevance of the model outputs that were validated to the model outputs used in the COU.

The V&V 40 framework requires that a credibility goal be defined for each credibility factor, ideally before data are collected. After establishing credibility goals, activities to achieve the credibility goals are planned and then executed to gather the V&V evidence (see Figure 2). Other pertinent information that supports credibility may include historical evidence of the model predictive ability for other COUs. It is then assessed whether the credibility goals were met, i.e., that the model is sufficiently credible for the COU. If the credibility goals are not achieved, then the COU or aspects of the model can be changed (e.g., to reduce the model risk and therefore the credibility requirements), or additional V&V activities can be performed. The final stage is to document the findings and include the rationale to support the assumptions and credibility goals for using the model in the proposed COU.

The ASME V&V 40 framework can serve as a guide for using CPMs in a variety of research roles such as hypothesis generation. The primary question of how it applies to questions such as generating hypotheses for future experimental studies comes to how risks associated with inaccurate predictions in these scenarios are evaluated. In these scenarios, the model will typically be responsible for 100% of the decision made, but the decision consequence of an inaccurate prediction comes in terms of wasted experimental time and cost rather than patient safety, as we are considering in the current work. Depending on how a researcher values the risks associated with the hypothesis they will test, they can use those risks to determine credibility goals and follow the V&V 40 framework accordingly. They could also use the approach to determine what might be necessary for using that same model for potential future uses as well by reviewing each credibility factor. This may lead to better planning of future studies and the information that it will be necessary to gather to support the CPM.

### Application of V&V 40 to Computational Patient Models for Testing PCLC Medical Devices

**FIGURE 3:**
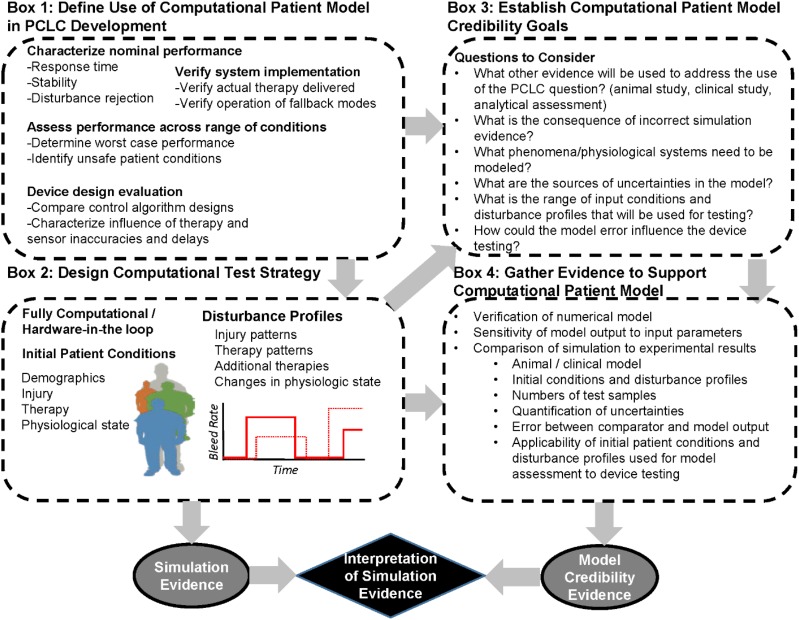
Workflow for generating evidence computational patient models during development of a physiological closed-loop controlled medical device using. The process begins with considering the question of interest (Box 1) that the computational patient model will be used to address. Next are the design of the computational test setup (Box 2) as well as establishment of the computational patient model credibility goals (Box 3). These are used to determine the evidence needed to support the use of the computational patient model (Box 4) for the particular question of interest. The evidence supporting the computational patient model credibility can be used to interpret the evidence from the computational device testing.

A simplified representation of how the V&V 40 framework may apply to the use of CPMs in PCLC testing is presented in Figure 3. The first steps of the V&V 40 framework are to define the question of interest and the COU of the physiological model. Box 1 of Figure 3 highlights several COUs we identified that computational testing has been used for in the design and evaluation of PCLC devices. These include confirming the performance of supervisory systems and fallback modes in the presence of known disturbances (artin et al., 1992) and assessing the controller performance to maintain a physiological variable within a certain range under a range of patient conditions (Bighamian et al., 2016). These two COUs may not require the same CPM or the same levels of evidence demonstrating CPM performance, as discussed in our recent case study examining how the ASME V&V 40 framework could be applied to evaluating automated fluid resuscitation systems (Scully et al., 2018). When assessing the system against disturbances, a simple model of the physiological system may be sufficient, but the realism of the different disturbance modes (such as signal noise in an arterial pressure waveform) will impact the credibility of the testing results. When representing a range of patient conditions, a more complex CPM may be needed resulting in assessment of the CPM across the range of physiologic conditions used for testing to ensure that the model produces realistic and expected results. These specific requirements should consider the question of interest being addressed (Box 3 in Figure 3). The V&V 40 framework requires an analysis of the risk associated with relying on the modeling results. For example, if test results from comprehensive animal studies are used alongside computational results to answer the question of interest, the influence of the model is lower and thus the credibility requirements may be lower. Alternatively, if results from computational testing alone will be used to make a high consequence decision, such as identifying a patient population a PCLC system is expected to safely be applied to in a clinical study, the credibility requirements for the model are expected to be higher.

In many of the articles reviewed, the CPM was used for both design and evaluation of controllers. Such overlapping roles for models with two distinctly different contexts of use requires the model to have credibility evidence for both uses. For example, the model needs to be simple and transparent enough to lend itself to controller design and at the same time be comprehensive enough to include the uncertainty and frequently occurring disturbances experienced by the patient and system. As models for design of controllers are often linearized, using the same model to challenge the controller and evaluate its safety may not be meaningful since the physiological process that the controller will be faced with is likely highly non-linear. Table 2 provides examples of how use of a computational model for design and evaluation may lead to different credibility goals when following the credibility framework.

**Table 2:** Application of the ASME V&V 40 risk-informed credibility framework to two different scenarios using computational models in the development of physiological closed-loop controlled medical devices.

		Scenario 1	Scenario 2
**Question of interest**		Design a controller to keep the physiological variable within X% of a set-point	Is a controller stable under variable patient conditions
**Context of use**		The controller will be synthesized by optimizing parameters to the computational patient model	The computational patient model will be used to perform a risk-based evaluation of the controller performance before being used in clinical studies.
**Model risk**	**Influence on decision**	High – no other evidence will be used to support the decision	High – no other evidence will be used to support the decision
	**Consequence of decision**	Low – following design of the controller, a series of studies including additional computational testing and animal studies will be performed to evaluate the controller performance before being used on patients	High – use of the controller on patients could lead to injury
	**Overall risk**	Medium	High
**Example credibility factors**		*Qualitative:* Parsimonious, low order, transparent, physiological relevant *Quantitative:* Linearizable, identifiable, predictive accuracy, reproduces uncertainty bounds	*Qualitative:* Transparent, physiologically relevant *Quantitative:* Identifiable, predictive accuracy, reproduces uncertainty bounds, generalizable
**Credibility activities**		Develop and perform plan to gather credibility evidence: experimental design, comparator (e.g., animal model), data analysis plan	


*The two scenarios outline example model risk and credibility factors. The noted model risk components including influence on decision and consequence of decision are different here to highlight how use of a model may influence the necessary evidence to support that use.*

One credibility factor assessed within the V&V 40 framework is the model form, that is, the overall structure of the model including the specific equations used. For a CPM, this includes the physiological systems, dynamic properties, and interactions between physiological systems that are modeled, together with the equations used to model those systems, properties and interactions. It may also include whether non-linearity or time-variance in parameters are considered. Much of this information may be supported by existing knowledge of physiology. The specific questions of the controller that will be addressed should be considered when determining the systems and properties important to the model. Uncertainty in the model form may come from physiological systems that are not modeled but known to interact with the system of interest, higher order dynamics that may have been linearized or simplified in the model, time-varying changes associated with physiological conditions or treatments, or stochastic properties in natural systems. Because of the myriad of possibilities that may influence the uncertainty of the model form, validation evidence comparing modeling results to independent experimental data to support that the model behaves as expected is a critical component of previous computational physiological model validation strategies (Cobelli et al., 1984; Hester et al., 2011). When developing credibility goals to support a specific use of a model, one should consider how these attributes may affect the specific testing results.

Model inputs is another credibility factor in the V&V 40 framework. For CPMs, this include parameters determining the physiological properties of the modeled systems, initial conditions, boundary conditions, and any external conditions including those provided by the PCLC device. When using a CPM to assess inter-patient variability, the range of initial conditions that the model produces valid results for should be established. The selection of parameters their sensitivity may impact the CPM. Parameter uncertainty during calibration or any data-driven fitting process may need to be evaluated. Initial conditions that may need to be considered include the current physiological state of the patient that may be affected by the amount of drug already given to the patient at the start of closed-loop therapy, for example. For closed-loop devices that are applied while patient physiological and clinical conditions may be changing, the loading conditions (disturbances) of the model and simulation need to be considered in relation to the dynamics of the system. For example, if system identification techniques are used to develop a data-driven model from experiments with a known input (e.g., a fixed hemorrhage followed by constant infusion of fluids), the performance of the model under different input conditions may not be captured (Bighamian et al., 2018). This could result in the need to compare the model to a variety of experimental conditions that are expected in the clinical scenario.

The final credibility factor considered here is the applicability of the V&V activities to the COU. In general, there are differences between how a model is validated and how it will be used (Pathmanathan et al., 2017), and for CPMs these differences can be considerable. For CPM with COUs geared toward PCLC testing, this may include species differences if animal experiments are used to validate the CPMs that will then be used for human physiology, or differences in patient populations if, for example, a healthy volunteer study is used to validate a model used to test a device that will be used on critically ill patients. Another difference might be the timescale of the validation tests as compared to the COU simulations. For example, validation tests that occur over a short time period may not adequately test whether the model replicates slow or delayed physiological changes that could be important in the COU simulations. Therefore, additional support can aid in establishing credibility of COU predictions given these differences between the validation and COU settings. For example, this may include physiological justification that validation using animal experiments is sufficient, considering the known differences of that species with humans. Other laboratory, clinical and experimental conditions can be contrasted to how the PCLC device will be used.

## Conclusion

Given the large body of work to advance control technology in other fields, the challenge for advancing PCLC medical devices in critical care medicine may not come from the availability of control strategies but instead result from sufficient understanding and modeling of patient physiology. There are fundamental questions with regards to the rigor of credibility evidence that need to be addressed to validate CPMs for design and evaluation of PCLC devices. With well-characterized and credible models, model-based strategies can be applied to design, iterate, and evaluate PCLC devices. Current evidence for demonstrating that a model is credible in PCLC development varies considerably, and rationales linking the evidence supporting a model for a specific use are limited in scope and presentation. Presenting this evidence, ideally by following a pre-specified framework, can aid in establishing the credibility of PCLC testing with CPMs.

## Disclosure

The mention of commercial products, their sources, or their use in connection with material reported herein is not to be construed as either an actual or implied endorsement of such products by the Department of Health and Human Services.

## Author Contributions

BP reviewed the articles related to anesthesia, hemodynamic/fluid resuscitation, and mechanical ventilation. He authored majority of these sections as well as the content of the discussion section. PP provided content on uncertainty quantification and model validation framework, and also reviewed the anesthesia section. CD reviewed the anesthesia section. FY reviewed the mechanical ventilation section. RG provided content on model validation. SW reviewed the mechanical ventilation section. TM reviewed the hemodynamic stability section and provided content on ASME V&V 40. CS provided content on hemodynamic stability and discussion section.

## Conflict of Interest Statement

The authors declare that the research was conducted in the absence of any commercial or financial relationships that could be construed as a potential conflict of interest.

## Footnotes

1 As a point of clarification, two models with the same equations but different values of model parameters are considered to have the same model form.

## Abbreviations

CO: cardiac output
COU: context of use
CPM: computational patient model
EtCO_2_: end-tidal partial pressure of carbon dioxide
MAP: mean arterial pressure
PCLC: physiological closed-loop control
PID: proportional-integral-derivative
PK/PD: pharmacokinetic-pharmacodynamic
SNP: sodium nitroprusside
V&V: verification and validation
